# A Ferroptosis-Related Gene Signature Identified as a Novel Prognostic Biomarker for Colon Cancer

**DOI:** 10.3389/fgene.2021.692426

**Published:** 2021-07-01

**Authors:** Xin Qi, Rui Wang, Yuxin Lin, Donghui Yan, Jiachen Zuo, Jiajia Chen, Bairong Shen

**Affiliations:** ^1^School of Chemistry and Life Sciences, Suzhou University of Science and Technology, Suzhou, China; ^2^Department of Urology, The First Affiliated Hospital of Soochow University, Suzhou, China; ^3^Institute for Systems Genetics, West China Hospital, Sichuan University, Chengdu, China

**Keywords:** colon cancer, ferroptosis, prognostic biomarker, gene signature, outcome risk nomogram

## Abstract

**Background:**

Colon cancer (CC) is a common gastrointestinal malignant tumor with high heterogeneity in clinical behavior and response to treatment, making individualized survival prediction challenging. Ferroptosis is a newly discovered iron-dependent cell death that plays a critical role in cancer biology. Therefore, identifying a prognostic biomarker with ferroptosis-related genes provides a new strategy to guide precise clinical decision-making in CC patients.

**Methods:**

Alteration in the expression profile of ferroptosis-related genes was initially screened in GSE39582 dataset involving 585 CC patients. Univariate Cox regression analysis and LASSO-penalized Cox regression analysis were combined to further identify a novel ferroptosis-related gene signature for overall survival prediction. The prognostic performance of the signature was validated in the GSE17536 dataset by Kaplan-Meier survival curve and time-dependent ROC curve analyses. Functional annotation of the signature was explored by integrating GO and KEGG enrichment analysis, GSEA analysis and ssGSEA analysis. Furthermore, an outcome risk nomogram was constructed considering both the gene signature and the clinicopathological features.

**Results:**

The prognostic signature biomarker composed of 9 ferroptosis-related genes accurately discriminated high-risk and low-risk patients with CC in both the training and validation datasets. The signature was tightly linked to clinicopathological features and possessed powerful predictive ability for distinct clinical subgroups. Furthermore, the risk score was confirmed to be an independent prognostic factor for CC patients by multivariate Cox regression analysis (*p* < 0.05). Functional annotation analyses showed that the prognostic signature was closely correlated with pivotal cancer hallmarks, particularly cell cycle, transcriptional regulation, and immune-related functions. Moreover, a nomogram with the signature was also built to quantify outcome risk for each patient.

**Conclusion:**

The novel ferroptosis-related gene signature biomarker can be utilized for predicting individualized prognosis, optimizing survival risk assessment and facilitating personalized management of CC patients.

## Introduction

Colon cancer (CC) is one of the most common gastrointestinal malignancies and the leading cause of cancer-related death worldwide (5.8%), accounting for over 1.09 million new cases and about 551,269 deaths in 2018 (Bray et al., 2018). Because of insidious onset and invasive rapid-progression, the majority of CC patients were diagnosed at advanced stages, thereby missing the optimal therapeutic regimens and opportunity. Moreover, the five-year and overall survival rate of CC patients remain unsatisfactory due to distant metastasis, recurrence and/or drug resistance following treatment (Goldstein et al., 2016; Van der Jeught et al., 2018; Leon et al., 2019). Currently, in clinical practice, treatment decision making for individual CC patients are mainly based on cancer- and patient-specific factors, such as gender, age and tumor-node-metastasis (TNM) staging. Nevertheless, due to high clinical heterogeneity of CC, those conventional clinical indicators are insufficient for accurate prediction of individualized prognosis. Therefore, establishing novel and robust predictive signatures that can reliably estimate clinical outcomes would have tremendous value in monitoring personalized prognosis and guiding clinical management of CC patients (Chen et al., 2013; Zhang et al., 2019; Liu et al., 2020b; Qi et al., 2020a).

Ferroptosis is a newly discovered iron-dependent form of regulated cell death (RCD) characterized by lipid peroxidation and accumulation of reactive oxygen species (ROS) (Angeli et al., 2019). Like other forms of RCD such as apoptosis and necroptosis, ferroptosis is strictly regulated under normal physiological conditions, and its dysregulation has been reported to be associated with a variety of pathological diseases (Stockwell et al., 2017). Notably, growing evidence has demonstrated that ferroptosis plays a pivotal role in cancer biology, making it an effective, prospective pathway for cancer therapy (Xu et al., 2019). For example, Xu et al. (2020) unveiled that colorectal cancer (CRC) stem cells are sensitive to ferroptosis, and inducing ferroptosis could attenuate the progression of those stem cells by modulating SLC7A11. Park et al. (2018) found that the activation of ferroptosis by bromelain effectively inhibit Kras-mutant CRC cells by up-regulating ACSL-4 expression. Similarly, Xia et al. (2020) discovered that talaroconvolutin A is a novel ferroptosis inducer with higher anticancer activity than erastin. It can suppress the growth of CRC cells via triggering ferroptosis. In addition, many ferroptosis regulators or markers such as GPX4 (Seibt et al., 2019), P53 (Jiang et al., 2015), RSL3 (Sui et al., 2018), Frataxin (Du et al., 2020), and DPP4 (Tang and Kroemer, 2020) are tightly linked to the initiation and progression of multiple cancer types, highlighting their novel potentials as cancer biomarkers. Given the important role of ferroptosis-related genes (FRGs) in cancer progression, ferroptosis-derived gene signatures for survival prediction have been successfully constructed in multiple cancer types, such as glioma (Liu et al., 2020a), hepatocellular carcinoma (Du and Zhang, 2020; Liang et al., 2020) and clear cell renal cell carcinoma (Wu et al., 2020). However, no ferroptosis-based prognostic model is available for CC that can be employed to predict patient clinical outcomes.

**FIGURE 1:**
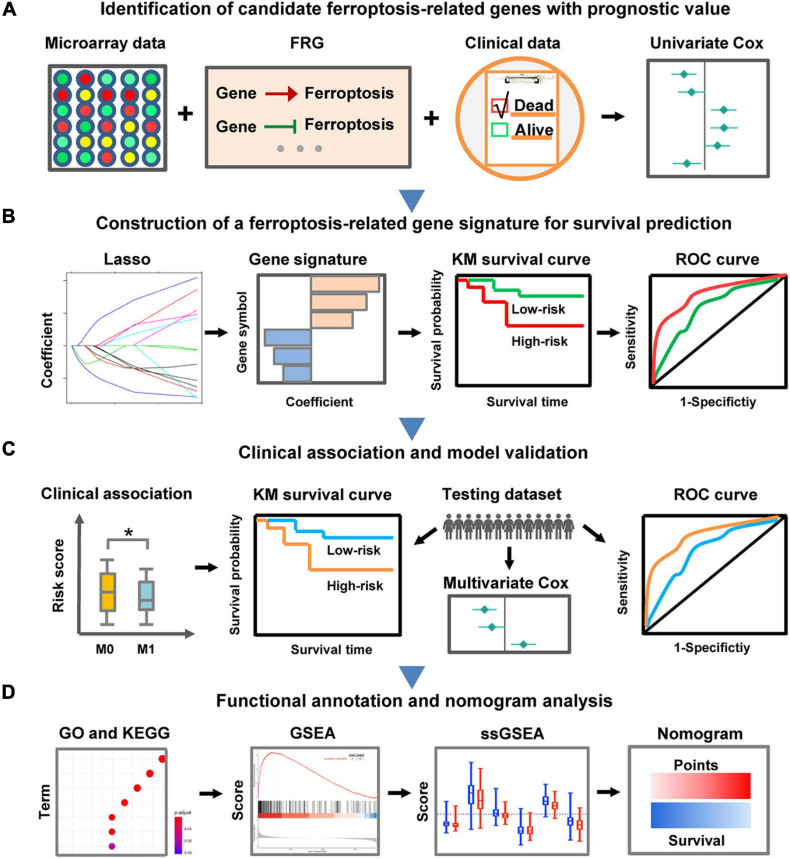
Schematic diagram of the FRG-related prognostic signature construction and characterization. **(A)** The FRGs with prognostic value were identified in CC through differential expression analysis and univariate Cox regression analysis. **(B)** LASSO Cox regression analysis was employed to develop a robust FRG signature for prognosis. **(C)** Clinical relevance exploration and independent validation of the FRG signature. The symbol ^∗^ means statistically significant. **(D)** Functional annotation of the FRG signature and construction of a nomogram predicting overall survival.

In the present study, we focused on decoding the prognostic implications of ferroptosis in CC by analyzing the transcriptome and clinical information retrieved from large-scale publicly available datasets. The pipeline of the present study was illustrated in Figure 1. Firstly, FRG with prognostic value in CC was identified via differential expression analysis and univariate Cox regression analysis. Next, a novel FRG-based signature for risk stratification and survival prediction was established by utilizing Lasso-penalized Cox regression analysis, and the association between the risk signature and clinicopathological characteristics was also investigated. Importantly, the prognostic performance of the FRG-based individualized signature was validated in another independent dataset by employing a series of bioinformatics approaches. Furthermore, a nomogram was built based on independent prognostic factors to quantify overall survival probability for patients with CC.

## Materials and Methods

### Acquisition of Ferroptosis-Related Gene

Ferroptosis-related genes (FRGs) that drive, suppress or mark ferroptosis were retrieved from the public FerrDb database^1^ (Zhou and Bao, 2020). After removing the duplicates, 260 FRGs were finally obtained for the following analyses (Supplementary Table 1).

### Data Collection and Identification of Differentially Expressed Genes

To develop a prognostic prediction model for patients with CC, the training and validation datasets containing expression profiles and clinical information were retrieved from the publicly available Gene Expression Omnibus^2^ (GEO) with the accession number GSE39582 (Marisa et al., 2013) and GSE17536 (Smith et al., 2010), respectively. Among them, the training dataset involves 585 patients, and the testing dataset contains 177 patients. The clinicopathological data for the samples collected in the current study were shown in Table 1. The “limma” R package for microarray data was then employed to identify differentially expressed genes (DEGs) between tumor and normal samples in the training cohort (Ritchie et al., 2015). The mRNAs that meet the defined criteria: fold change > 1.5 or < −0.67 and adjusted *p* < 0.05, were considered as up-regulated or down-regulated DEGs.

**TABLE 1:** Clinical features of the CC patients involved in this study.

	GSE39582	GSE17536
No. of patients	549	175
Age (median, range)	68 (22–97)	66 (26–92)
**Gender (%)**		
Female	247 (44.99%)	80 (45.71%)
Male	302 (55.01%	95 (54.29%)
**Status (%)**		
Alive	364 (66.30%)	103 (58.86%)
Dead	185 (33.70%)	72 (41.14%)
OS days (median)	1311.7	1560
**Clinical stage (%)**		
0	4 (0.73%)	0
1	31 (5.65%)	24 (13.71%)
2	256 (46.63%)	57 (32.57%)
3	200 (36.43%)	56 (32.00%)
4	58 (10.56%)	38 (21.71%)
**T stage (%)**		
T0	1 (0.18%)	N/A
T1	11 (2.00%)	N/A
T2	42 (7.65%)	N/A
T3	355 (64.66%)	N/A
T4	117 (21.31%)	N/A
Tis	3 (0.55%)	N/A
N/A	20 (3.64%)	N/A
**N stage (%)**		
N+	6 (1.09%)	N/A
N0	291 (53.01%)	N/A
N1	128 (23.32%)	N/A
N2	98 (17.85%)	N/A
N3	6 (1.09%)	N/A
N/A	20 (3.64%)	N/A
**M stage (%)**		
M0	468 (85.25%)	N/A
M1	59 (10.75%)	N/A
MX	2 (0.36%)	N/A
N/A	20 (3.64%)	N/A
**Tumor location (%)**		
Distal	333 (60.66%)	N/A
Proximal	216 (39.34%)	N/A

### Establishment of the FRG-Based Prognostic Signature

To construct the FRG-based prognostic prediction model, univariate Cox analysis was firstly carried out using the coxph function of the “survival” R package to screen differentially expressed FRGs (DE-FRGs) that significantly associated with overall survival (OS) of CC patients (Therneau, 2020). To obtain robust prognostic genes, samples with incomplete clinical data and OS less than or equal 1 month in GSE39582 dataset were removed. The expression level of genes was normalized by the “scale” function in R. Normalized expression data frame of DE-FRGs and prognostic data including OS time and OS status were used as the input files in the univariate Cox regression analysis. The DE-FRGs with *p*-values less than 0.05 were retained as candidate genes with prognostic value. Thereafter, to further minimize over-fitting effect, the LASSO-penalized Cox regression analysis was performed to construct the FRG-based prognostic model using the “glmnet” R package (Friedman et al., 2010). The minimum λ was determined through 10-fold cross-validation. According to the normalized expression value and coefficient of each FRG that composed the prognostic signature, the risk score of each included patient was calculated by the following formula:

(1) $$Risk\;score=\sum_{i=1}^{n}Coef_{i}\times x_{i},$$

Where n represents the number of genes that make up the model, Coef equals the coefficient of each prognostic FRG, x is the normalized expression level of each prognostic FRG. Then, the regression coefficients from the training dataset were applied into the validation dataset to compute risk scores. Further, multivariate Cox regression analysis for the risk score and clinical factors was conducted to measure whether the FRG-based signature was an independent prognostic indicator for OS.

### Kaplan-Meier Survival Analysis and ROC Analysis of the Prognostic Model

To evaluate the prognostic performance of the established FRG signature, the patients in each dataset were divided into high-risk and low-risk groups. Then, Kaplan-Meier survival curve analyses were employed to determine whether there was a significant difference in OS time between high-risk and low-risk groups using the “survival” and “survminer” R packages (Alboukadel et al., 2018; Therneau, 2020). And the log-rank test (*p* < 0.05) was used to measure the difference. Furthermore, time-dependent ROC curve analysis was performed with “timeROC” and “survival” R packages to assess the predictive power of the prognostic signature (Blanche, 2015; Therneau, 2020).

### Functional Enrichment Analysis of the Prognostic Signature

To decode the function of the FRG-based signature in CC, DEGs between the high-risk and low risk groups were identified with the criteria (fold change > 1.5 or < −0.67 and adjust *p* < 0.05). Then, Gene Ontology (GO) and Kyoto Encyclopedia of Genes and Genomes (KEGG) pathway enrichment analyses of the signature-related DEGs were performed utilizing the “clusterProfiler” R package (v3.14.3) (Yu, 2018). *P*-values adjusted by Benjamini-Hochberg method (adj. *p-*value < 0.05) were employed to select statistically significant GO or KEGG terms. Furthermore, gene set enrichment analysis (GSEA)^3^ was carried out to interpret the enriched pathways of the identified DEGs between the high-risk and low risk groups. MSigDB gene set “h.all.v7.2.entrez.gmt” that represents well-defined biological states or processes was derived from the Molecular Signatures Database^4^. The “enrichplot” R package was utilized to exhibit the enriched GSEA pathways. Moreover, single-sample gene set enrichment analysis (ssGSEA) was performed with “GSVA” R package to determine the variation in immune-related biological processes between high-risk and low-risk groups (Hänzelmann et al., 2013). The well-defined gene set was downloaded from the reported reference (Liang et al., 2020).

### Nomogram Analysis

According to the findings from the multivariate Cox regression analysis, the “rms” R package was utilized to build the nomogram based on independent prognostic factors including risk score, age and stage (Zhang and Kattan, 2017). The predictive power of the nomogram was evaluated by using the calibration curves and the concordance index (C-index) value.

### Statistical Analysis

All statistical analyses were conducted using the R software (Version 3.6.2) or GraphPad Prism (Version 9.0.0). If not specified, a two-sided *p*-value or adjusted *p*-value less than 0.05 was considered statistically significant.

## Results

### Identification of CC-Specific Prognostic Genes Involved in Ferroptosis

**FIGURE 2:**
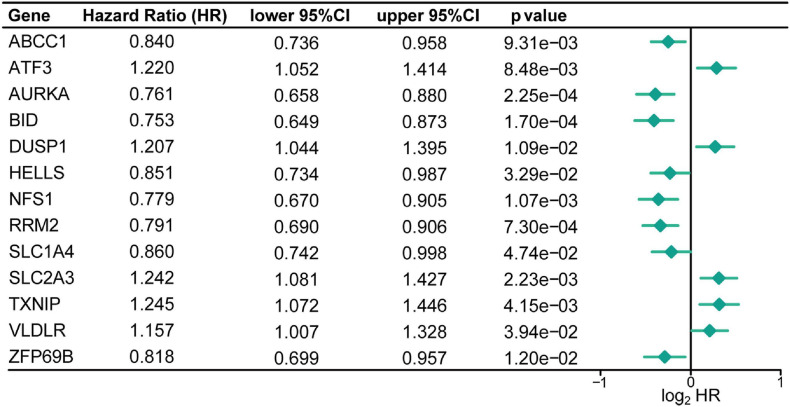
Identification of the FRGs with prognostic value in patients with CC. Forest plots shows the differentially expressed FRGs identified by univariate Cox regression analysis that can predict overall survival in patients with CC.

To investigate FRGs with prognostic roles in CC, 260 FRGs were firstly collected from FerrDb, a database for ferroptosis regulators and markers. Through differential expression analysis, 68 FRGs (38 up-regulated and 30 down-regulated) were then identified as significantly dysregulated genes in CC specimens compared with control tissues in GSE39582 dataset (Supplementary Figure 1). Subsequently, univariate Cox regression analysis of those differentially expressed FRGs showed that the expression level of 13 FRGs, namely *ABCC1*, *ATF3*, *AURKA*, *BID*, *DUSP1*, *HELLS*, *NFS1*, *RRM2*, *SLC1A4*, *SLC2A3*, *TXNIP*, *VLDLR*, and *ZFP69B*, were significantly related to CC overall survival (Figure 2 and Supplementary Table 2). Besides, CC patients were classified into 6 molecular subtypes in the GSE39582 cohort. Considering the impact of molecular subtypes on the prognosis of CC patients, the subtype-based differential expression analysis was also carried out. As shown in Supplementary Table 3, most of the identified 13 DE-FRGs with prognostic value was significantly differentially expressed in the six comparison groups, respectively, highlighting their important roles in CC.

### Establishment of a Ferroptosis-Related Gene Signature for Prognosis Prediction in CC Patients

**FIGURE 3:**
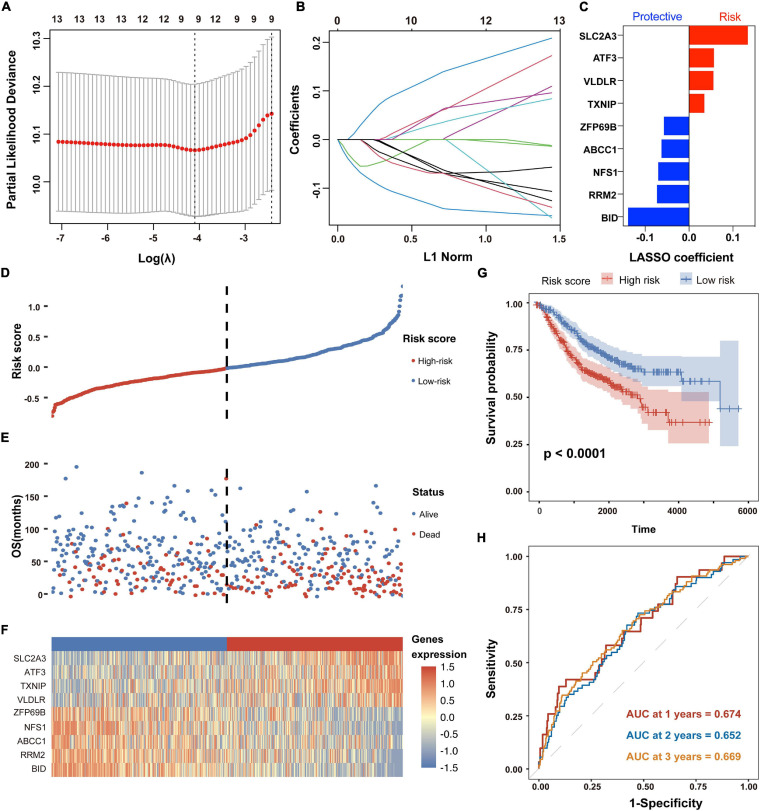
Establishment and prognostic analysis of the FRG signature in CC. **(A,B)** The LASSO Cox regression analysis was employed to identify the most robust prognostic FRGs, with the optimal λ value of 0.0166. **(C)** Distribution of LASSO Cox coefficients of nine FRGs that constitute the prognostic signature. **(D)** Distribution of risk scores based on the FRG prognostic signature in the GSE39582 dataset. **(E)** Survival status of CC patients with high or low risk scores in the GSE39582 dataset. **(F)** Heatmap shows the expression pattern of nine FRGs that constitute the prognostic signature in the GSE39582 dataset. **(G)** Kaplan-Meier plot for overall survival of CC patients in high- and low-risk groups in the GSE39582 dataset. **(H)** Time-dependent ROC curves for the prognostic performance of the FRG signature in the GSE39582 dataset.

To construct a FRG-based prognostic signature for predicting overall survival in CC patients, we performed LASSO Cox regression analysis to screen out the most robust prognostic genes from 13 candidate FRGs. According to the optimal value of λ, a 9-FRG signature was finally established (Figures 3A–C). For each patient in the GSE39582 dataset, a risk score was calculated based on the regression coefficient and expression level of each FRG. Accordingly, the CC patients were stratified into high-risk and low-risk groups according to the median value of risk scores (Figure 3D). For each CC patient, the higher risk score indicated a poor overall survival. Therefore, the FRGs with a higher expression level and larger positive coefficient tend to have an increased risk feature of death, and vice versa.

As shown in the survival status distributions of Figure 3E, high-risk patients were more likely to have shorter survival time compared with those low-risk patients. Expression profile of the 9 FRGs that constitute the prognostic signature was obviously altered between high- and low-risk patients with CC (Figure 3F). Consistently, the Kaplan-Meier survival analysis demonstrated that patients with high risk scores exhibited significantly worse OS in the GSE39582 dataset (*p* < 0.05, Figure 3G). According to the results of time-dependent ROC curve analyses, the area under the curve (AUC) value reached 0.674 at 1 year, 0.652 at 2 years and 0.669 at 3 years, respectively (Figure 3H). Besides, time-dependent ROC analysis was also performed in subgroups of TNM stages. As shown in Supplementary Figure 2, the signature exhibited better performance in stage I (AUC value > 0.890) and stage III (AUC value > 0.679) than that in all patients. These results suggested that the FRG-derived prognostic signature is closely associated with OS of CC patients.

### Ferroptosis-Related Gene Signature Is Closely Related to Clinicopathological Features

**FIGURE 4:**
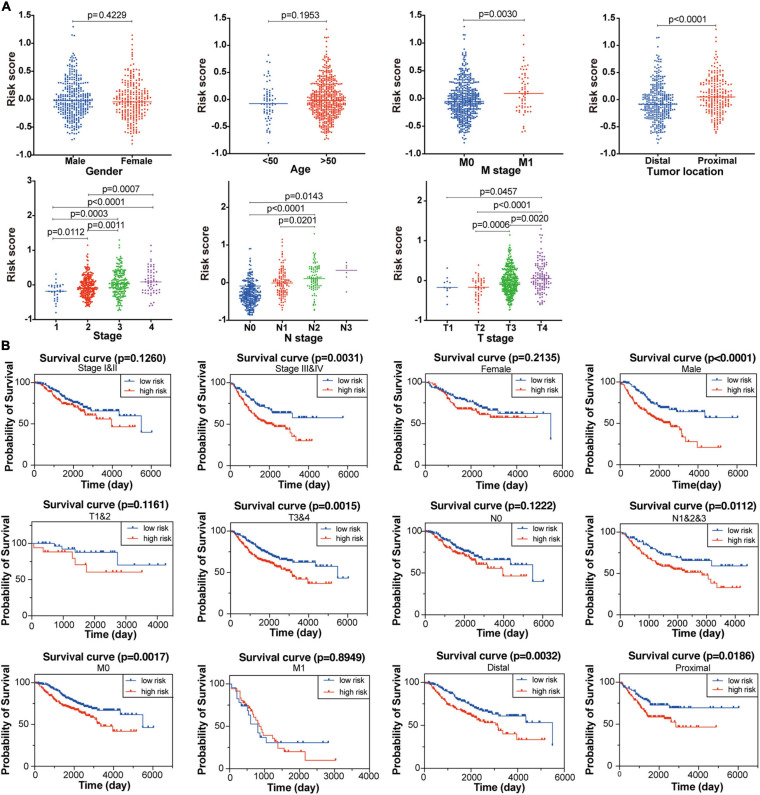
Association between the established FRG signature and clinicopathological characteristics. **(A)** Correlation analyses of risk scores calculated based on the FRG signature with multiple clinicopathological features of CC patients in the GSE39582 dataset, including gender, age, stage, tumor location, T stage, N stage and M stage. Statistical significance was calculated using the Student’s *t*-test (*p* < 0.05). **(B)** Subgroup Kaplan-Meier curve analysis shows overall survival probability of high- and low-risk CC patients from the GSE39582 dataset stratified by stage (stage I/II vs. stage III/IV), gender (female vs. male), T stage (T1/2 vs. T3/4), N stage (N0 vs. N1/2/3), M stage (M0 vs. M1), and tumor location (distal vs. proximal). Statistical significance was calculated using the log-rank test (*p* < 0.05).

To explore the association between the established signature and clinicopathological characteristics, we compared the risk scores in different subgroups stratified by age, sex, tumor location, TNM stage, T stage, N stage, and M stage, respectively. As shown in Figure 4A, the risk score of patients with advanced CC was significantly higher than that of patients with primary CC. For tumor location, the risk scores obviously decreased in patients with distal tumor location compared with those with primary tumor location. However, the risk score was not significantly associated with age and gender. These results revealed that the established FRG-based prognostic signature was closely related to clinicopathological features including tumor location, TNM stage, T stage, N stage, and M stage.

Furthermore, to better evaluate the prognostic performance of the constructed signature, we conducted a stratified survival analysis based on clinical risk factors to examine whether it retains the robust ability to predict overall survival of distinct subgroups. Within each subgroup of TNM stage (stage I/II and stage III/IV), tumor location (primary and distal), T stage (T1/2 and T3/4), N stage (N0 and N1/2/3), M stage (M0 and M1), the CC patients were divided into high-risk and low-risk groups according to the median risk score calculated through the constructed formula. As shown in Figure 4B, patients with high-risk score had significantly poor survival probability than those with low-risk score in most of the above subgroups, indicating the signature’s powerful predictive capacity.

### Prognostic Validation of the Ferroptosis-Related Gene Signature in Independent Dataset

**FIGURE 5:**
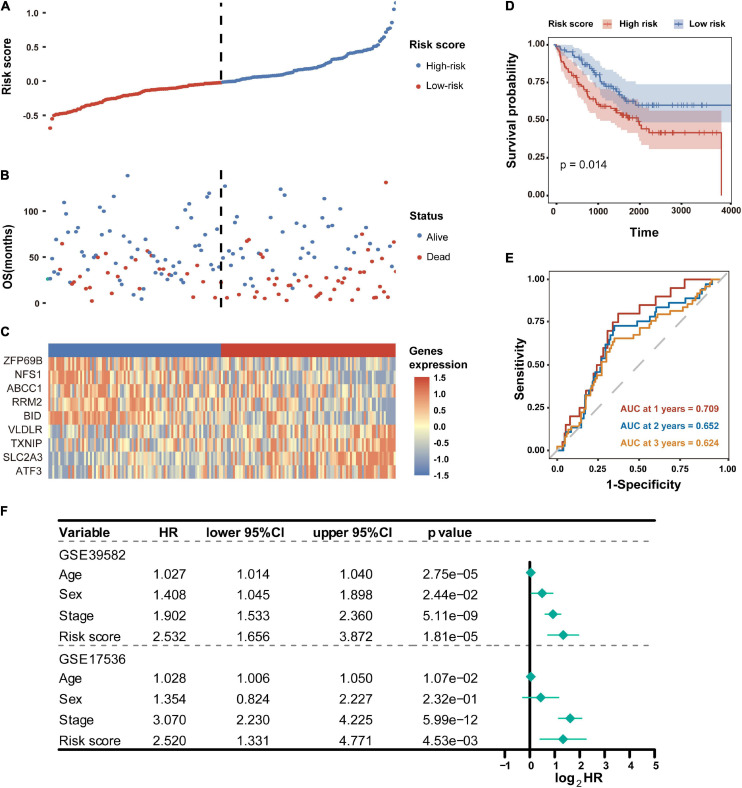
Validation of the FRG prognostic signature in the GSE17536 dataset. **(A)** Distribution of risk scores based on the FRG prognostic signature in the GSE17536 dataset. **(B)** Survival status of CC patients with high or low risk scores in the GSE17536 dataset. **(C)** Heatmap shows the expression pattern of nine FRGs that constitute the prognostic signature in the GSE17536 dataset. **(D)** Kaplan-Meier plot for overall survival of CC patients in high- and low-risk groups in the GSE17536 dataset. **(E)** Time-dependent ROC curves for the prognostic performance of the FRG signature in the GSE17536 dataset. **(F)** Multivariate Cox regression analyses evaluated the prognostic independence of the FRG signature regarding overall survival in GSE39582 and GSE17536 datasets. HR, hazard ratio.

To test the robustness of the established prognostic model, CC patients from the GSE17536 cohort were divided into high-risk and low-risk groups by the median risk score value calculated with the formula constructed from the GSE39582 dataset (Figure 5A). Consistently, patients in the high-risk group tended to death earlier (Figure 5B) and had shorter overall survival time than their low-risk counterparts (Figures 5D,E). As shown in the heatmap, the expression abundance of 9 genes in the model was significantly different between high- and low-risk patients (Figure 5C). These results indicated that the FRG-based prognostic signature had a robust and stable power in predicting OS of CC patients. In addition, SLC2A3 and NFS1 were used as representative FRGs to verify their expression pattern between CRC and normal control tissues. As shown in Supplementary Figure 3, the expression level of SLC2A3 and NFS1 were significantly higher in CRC tissues compared with that in normal colon tissues.

To further determine whether the FRG-based prognostic signature can act as an independent overall survival predictor, multivariate Cox regression analyses were carried out using available clinical factors and risk score as variables. As shown in Figure 5F, the risk score was dramatically correlated with OS of patients in both the GSE39582 and GSE17536 cohorts, revealing that the risk score proved to be an independent prognostic factor for CC patients.

### Functional Annotation for the Ferroptosis-Related Gene Signature in CC

**FIGURE 6:**
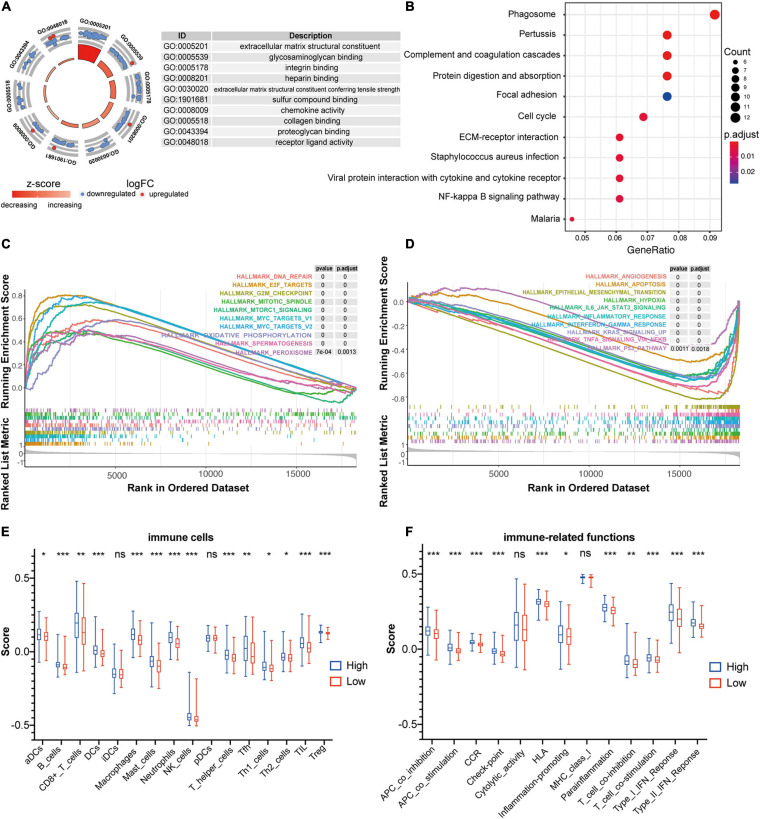
Functional annotation of the FRG prognostic signature. **(A)** Circle plot shows the top ten enriched GO terms for the DEGs identified between high- and low-risk groups in the training dataset of GSE39582. **(B)** Bubble diagram shows the enriched KEGG pathways for the DEGs identified between high- and low-risk groups in the training dataset of GSE39582. **(C,D)** GSEA analysis was used to explore cancer hallmarks that enriched in the high-risk subgroup **(C)** and low-risk subgroup **(D)** of the GSE39582 dataset. **(E)** Comparison of the ssGSEA scores of 16 immune cells between high- and low-risk groups in the GSE39582 dataset. Statistical significance was calculated using the Student’s *t*-test (*p* < 0.05). *p < 0.05; **p < 0.01; ***p < 0.001. **(F)** Comparison of the ssGSEA scores of 13 immune-related functions between high- and low-risk groups in the GSE39582 dataset. Statistical significance was calculated using the Student’s *t*-test (*p* < 0.05). *p < 0.05; **p < 0.01; ***p < 0.001.

To gain insights into the biological features implicated with the FRG-based prognostic signature, functional enrichment analyses including GO and KEGG, were conducted based on the DEGs identified between high- and low-risk groups in the training dataset. As shown in Figure 6A, the DEGs were significantly enriched in GO terms, such as extracellular matrix (ECM) structural constituent, chemokine activity, receptor ligand activity, and binding of proteoglycan, integrin, heparin, sulfur compound, and collagen. Consistently, chemokine signaling has been demonstrated to be associated with ferroptosis in hepatocellular carcinoma (Liang et al., 2020). As expected, those DEGs were strikingly involved in the well-characterized pathways associated with CC including cell cycle, NF-κB signaling pathway, ECM-receptor interaction, etc. (Figure 6B).

Moreover, GSEA analyses were performed to compare the distinct pathways between high-risk and low risk groups of the FRG-based prognostic signature. As shown in Figure 6C, multiple cancer-associated hallmarks, e.g., DNA repair, G2M checkpoint and mitotic spindle that related to cell cycle, oxidative phosphorylation and peroxysome that associated with ferroptosis, and E2F targets, mTORC1 signaling and MYC targets that linked to transcriptional regulation, were highly enriched in CC patients with a high-risk score. Comparatively, the low-risk group was significantly associated with multiple cancer-related processes, such as angiogenesis, apoptosis, epithelial-mesenchymal transition, hypoxia, IL6_JAK_STAT3 signaling, inflammatory response, interferon γ response, KRAS signaling, TNFa signaling via NF-κB, and P53 pathway (Figure 6D).

In addition, given the pivotal role of immune in tumor pathogenesis, the ssGSEA enrichment analysis was employed to further explore the association between prognostic signature risk score and immune-related cell type or functions. As illustrated in Figure 6E, the score of multiple immune cell types such as aDCs, B cells, CD8^+^ T cells, DCs, iDCs, NK cells, TIL, Treg, and etc., were significantly higher in high-risk group than that in low-risk group. Notably, the high-risk group was dramatically enriched in critical immune events or processes compared with the low-risk counterpart, e.g., checkpoint, HLA, IFN response, inflammation-promoting, and etc. (Figure 6F). Therefore, these results provided valuable insights for individualized treatments of CC patients with different risk scores.

### Nomogram Construction Based on FRG Prognostic Signature for Personalizing Overall Survival Prediction in CC Patients

**FIGURE 7:**
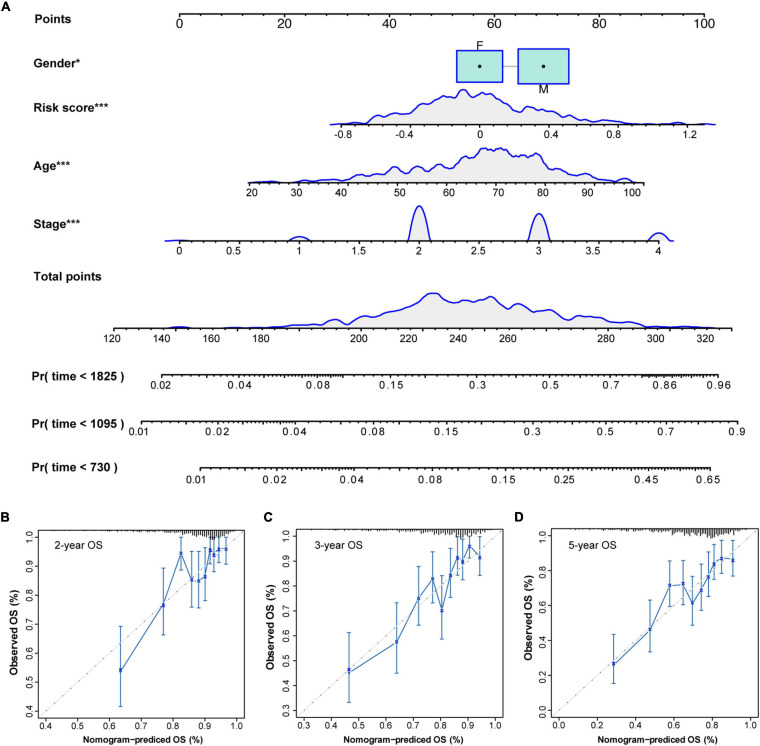
A nomogram for predicting the probability of patient overall survival based on FRG signature and clinical factors. **(A)** A nomogram with FRG signature and clinical variables was built to quantify survival probability for individual CC patient. Survival time is measured in days. The independent prognostic factors including risk score, gender, age and stage were selected from multivariate Cox regression analysis. *p < 0.05; ***p < 0.001. **(B–D)** Calibration curves of the nomogram for predicting the 2-year **(B)**, 3-year **(C)**, and 5-year **(D)** survival probability. Closer alignment with the 45-degree line represents a better performance.

To develop a clinical method for quantifying the risk assessment and overall survival probability for individual CC patient, a nomogram with independent prognostic factors including the FRG signature, sex, age, and stage, was constructed based on the result of multivariate analysis in GSE39582 dataset (Figure 7A). The larger total points in the nomogram indicated a shorter overall survival probability. The C-index of the nomogram was 0.694 (95% CI, 0.655–0.733), and the calibration curves showed a good agreement between nomogram prediction and actual outcomes in the probability of 2-year, 3-year, and 5-year survival (Figures 7B–D), suggesting a powerful predictive capacity of the nomogram.

## Discussion

The discovery of RCD processes has enabled striking advances in cancer intervention and therapeutics. As a novel RCD form, ferroptosis has emerged to play a crucial role in cancer development and treatment response, making it a promising biomarker and effective target in cancer therapy. Accordingly, ferroptosis gene signatures have been built for survival prediction in several types of cancer, e.g., glioma (Liu et al., 2020a), hepatocellular carcinoma (Du and Zhang, 2020; Liang et al., 2020) and clear cell renal cell carcinoma (Wu et al., 2020). However, the prognostic role of ferroptosis gene signature has yet to be explored in CC. Therefore, we herein aimed to establish a FRG-based signature for risk stratification and survival prediction in CC patients and decode its prognostic implications through comprehensive methods.

Firstly, alteration of FRG expression profile was initially determined in transcriptomic dataset involving 585 patients. To our excitement, 9 FRGs (*SLC2A3*, *ATF3*, *VLDLR*, *TXNIP*, *ZFP69B*, *ABCC1*, *NFS1*, *RRM2*, and *BID*) were identified as powerful prognostic indicators of overall survival in patients with CC by univariate Cox regression analysis as well as LASSO-penalized Cox regression analysis (Figure 3C). As reported, overfitting is a common characteristic of survival analysis modeling using microarray data (Xiong et al., 2020). Compared with Cox regression, Lasso-Cox regression can identify genes with high prognostic performance by minimizing the risk of overfitting, so it is widely used to construct survival prediction models for a variety of cancers (Liang et al., 2020; Wu et al., 2020). Therefore, the present study adopted the Lasso-Cox regression analysis to screen FRGs with prognostic value.

Notably, most of these FRGs have been identified as prognostic biomarkers and implicated in ferroptosis process of multiple types of tumors. For example, *SLC2A3* could act as a biomarker to determine prognosis and immune infiltration in gastric cancer by mediating glycolysis reprogramming (Yao et al., 2020). The stress response gene *ATF3*, has been shown to activate erastin-induced ferroptosis by repressing amino acid antiporter system Xc^–^ (Wang et al., 2020) and could serve as a prognostic biomarker in hepatocellular carcinoma (Li et al., 2021). *TXNIP* has been implicated in ferroptosis in retinal cells (Singh et al., 2020), and its downregulation indicates poor prognosis in patients with clear cell renal cell carcinoma (Gao et al., 2020). Furthermore, *ABCC1*, also known as multidrug resistance protein 1 (*MRP1*), was confirmed to affect ferroptosis process by mediating GSH efflux (Cao et al., 2019). As a regulator of iron metabolism in ferroptosis (Shi et al., 2019; Chen et al., 2020), *NFS1* has been demonstrated to reduce ferroptosis sensitivity (Dixon and Stockwell, 2019). *RRM2*, a regulator of DNA replication and repair, could protect liver cancer cells against ferroptosis (Yang et al., 2020). *BID*, a member of pro-apoptotic BCL2 family, plays a pivotal role in oxidative death (Kuang et al., 2020). Mitochondrial transactivation of *BID* could mediate ROS-induced ferroptosis (Neitemeier et al., 2017). In particular, as prognostic indicators, *ABCC1* and *NFS1* have also been included in the survival prediction model of hepatocellular carcinoma, implying their robust roles in survival prediction (Liang et al., 2020).

A novel prognostic signature composed of the above nine FRGs was then built for predicting overall survival of patients with CC. As expected, patients with high risk scores calculated using the FRG signature-based formula showed significantly poorer overall survival, which has been verified in another independent dataset (Figures 3G, 5D). The signature is closely related to clinicopathological features as shown in the stratified Kaplan-Meier plots (Figure 4B). Furthermore, the risk score was confirmed to be an independent prognostic factor for CC patients by multivariate Cox regression analysis Figure 5F). Therefore, these results revealed that the novel FRG signature has great potential in guiding personalized treatment for each CC patient.

Recent discoveries have shown that CD8^+^ T cells can promote tumor ferroptosis during immune checkpoint therapy with PD-L1 blockade, and suppression of ferroptosis inhibited the anti-tumor effect of PD-L1 blockade, revealing that T cell-induced tumor ferroptosis is an anti-tumor immunological mechanism (Stockwell and Jiang, 2019; Wang et al., 2019). Consistently, functional annotation of the 9-FRG signature showed that DEGs identified between high- and low-risk groups were significantly enriched in immune and inflammatory processes, such as ECM-related pathway, chemokine activity, cell cycle, and NF-κB signaling pathway (Figure 6B). Furthermore, the results of GSEA and ssGSEA analyses also indicated that the FRG prognostic signature is closely correlated with pivotal cancer hallmarks, particularly cell cycle, transcriptional regulation, and immune-related functions (Figures 6E,F). Thus, these findings highlight that the ferroptosis activation is a promising pathway for cancer immune intervention.

There are some limitations in this study, which should be further addressed in the future. First, the FRG-based prognostic signature was both constructed and validated in microarray dataset. Considering the impact of the data generation platform, the prognostic validation of the signature also needs to be performed in RNA-Seq-based dataset. As shown in Supplementary Figure 4, the signature that containing the 8 FRGs except VLDLR had a good prognostic performance in the CC cohort from TCGA database (TCGA-COAD). Due to low expression level, the VLDLR gene was filtered out of the signature during the Kaplan-Meier survival curve analysis and time-dependent ROC curve analysis of TCGA-COAD cohort, so its prognostic role in CC needs further research. Moreover, a better strategy is to construct signatures in the RNA-Seq-based and microarray-based datasets separately and further examine the prognostic performance of the overlapped multi-gene signature. Second, as the FRG prognostic signature was constructed and verified with retrospective cohorts from public available databases, its prognostic robustness and clinical utility need to be further validated in larger prospective trials. Third, the method for FRG selection only focused on the reported FRGs in the FerrDb database, possibly ignoring the role of other “unidentified” FRG or “indirect regulators” of ferroptosis. The approach that using 260 genes and not the entire genome for univariate cox analysis may lead to false positives and needs to be validated using wet lab techniques. Besides, further *in vitro* and *in vivo* experimental studies are required to provide deep insight into the biological roles of this FRG signature in CC.

In addition, increasing evidence has indicated that molecular features, risk factors and clinical outcomes for CRC may vary by anatomic subsites (e.g., colon and rectum) (Buchwald et al., 2018; Demb et al., 2019; Qi et al., 2020b). Therefore, whether the FRG signature established in CC is robust and stable for prognosis stratification in CRC will be the focus of our further research.

## Conclusion

In summary, our study established a novel prognostic FRG signature that performs well in individualized risk stratification and survival prediction for patients with CC. The signature is closely related to clinicopathological features and possesses powerful predictive ability for distinct clinical subgroups. Particularly, the FRG prognostic signature is closely linked to immune-related functions, implying the critical role of ferroptosis in immunotherapy. By integrating this signature with other independent clinicopathological factors, a nomogram was also built to quantify outcome risk for each patient. Therefore, the constructed FRG signature could be utilized for predicting individualized prognosis and facilitating personalized management of CC patients.

## Data Availability Statement

Publicly available datasets were analyzed in this study. This data can be found here: https://www.ncbi.nlm.nih.gov/geo/.

## Author Contributions

BS, XQ, and JC designed the research. XQ and RW collected the data and performed the computational analyses. XQ, RW, DY, and JZ drafted the manuscript. BS, XQ, YL, and JC revised the manuscript. All authors read and approved the final manuscript.

## Conflict of Interest

The authors declare that the research was conducted in the absence of any commercial or financial relationships that could be construed as a potential conflict of interest.

## Footnotes

1 http://www.zhounan.org/ferrdb/index.html

2 https://www.ncbi.nlm.nih.gov/geo

3 http://software.broadinstitute.org/gsea/downloads.jsp

4 http://software.broadinstitute.org/gsea/msigdb/index.jsp

## Supplementary Material

Supplementary Figure 1 Venn diagram shows the number of overlapping genes between DEGs and FRGs.

Supplementary Figure 2 Time-dependent ROC curves for the prognostic performance of the FRG signature by subgroups of TNM stages in the GSE39582 dataset.

Supplementary Figure 3 Protein expression level of SLC2A3 **(A)** and NFS1 **(B)** in normal colon and CC tissues based on immunohistochemistry images from the human protein atlas database. NC, normal colon; CC, colon cancer.

Supplementary Figure 4 Validation of the FRG prognostic signature in the TCGA-COAD dataset. **(A)** Kaplan-Meier plot for overall survival of CC patients in high- and low-risk groups. **(B)** Time-dependent ROC curves for the prognostic performance of the FRG signature. It should be noted that the *VLDLR* gene was filtered out from the signature during Kaplan-Meier survival curve analysis and time-dependent ROC curve analysis due to its low expression level in the TCGA-COAD dataset.

Supplementary Table 1 The FRGs retrieved from the public FerrDb database.

Supplementary Table 2 Differential expression pattern of prognostic FRGs between tumor and normal control tissues.

Supplementary Table 3 Subtype-based differential expression pattern of prognostic FRGs between tumor and normal control tissues.

## Abbreviations

CC: colon cancer
CRC: colorectal cancer
TNM: tumor-node-metastasis
RCD: regulated cell death
ROS: reactive oxygen species
FRG: ferroptosis-related gene
GEO: Gene Expression Omnibus
DEGs: differentially expressed genes
OS: overall survival
GO: Gene Ontology
KEGG: Kyoto Encyclopedia of Genes and Genomes
GSEA: gene set enrichment analysis
ssGSEA: single-sample gene set enrichment analysis
C-index: concordance index
AUC: area under the curve
ECM: extracellular matrix
MRP1: multidrug resistance protein 1.
