# Supplementary figures and images for: A Ferroptosis-Related Gene Signature Identified as a Novel Prognostic Biomarker for Colon Cancer
Source: Front Genet. 2021 Jul 1;12:692426. doi: 10.3389/fgene.2021.692426 (PMC8280527; doi:10.3389/fgene.2021.692426)

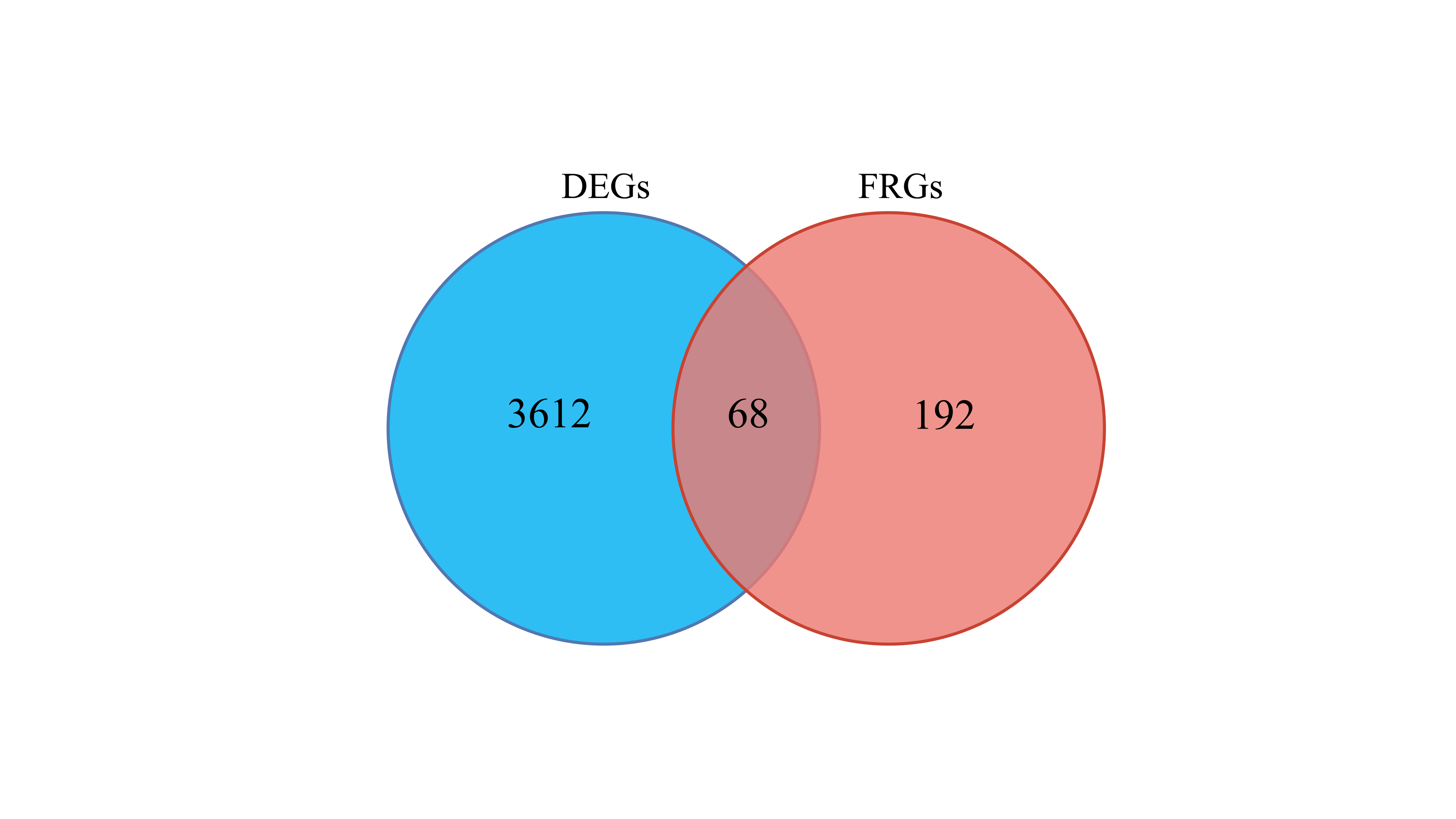

Supplement: Supplementary Figure 1 — Venn diagram shows the number of overlapping genes between DEGs and FRGs. [file Image_1.TIF]

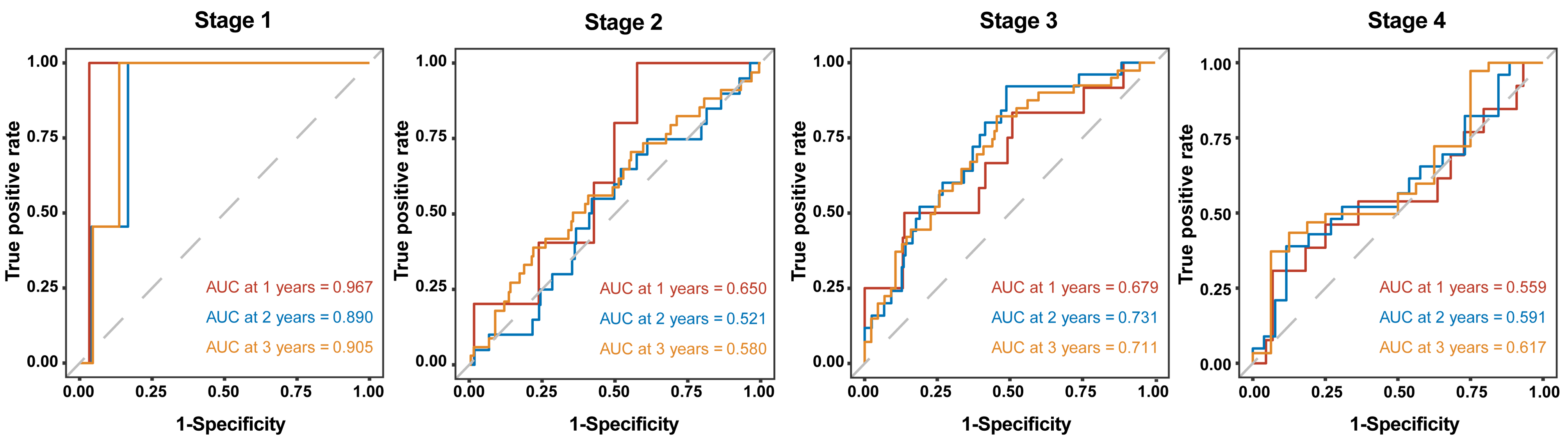

Supplement: Supplementary Figure 2 — Time-dependent ROC curves for the prognostic performance of the FRG signature by subgroups of TNM stages in the GSE39582 dataset. [file Image_2.TIF]

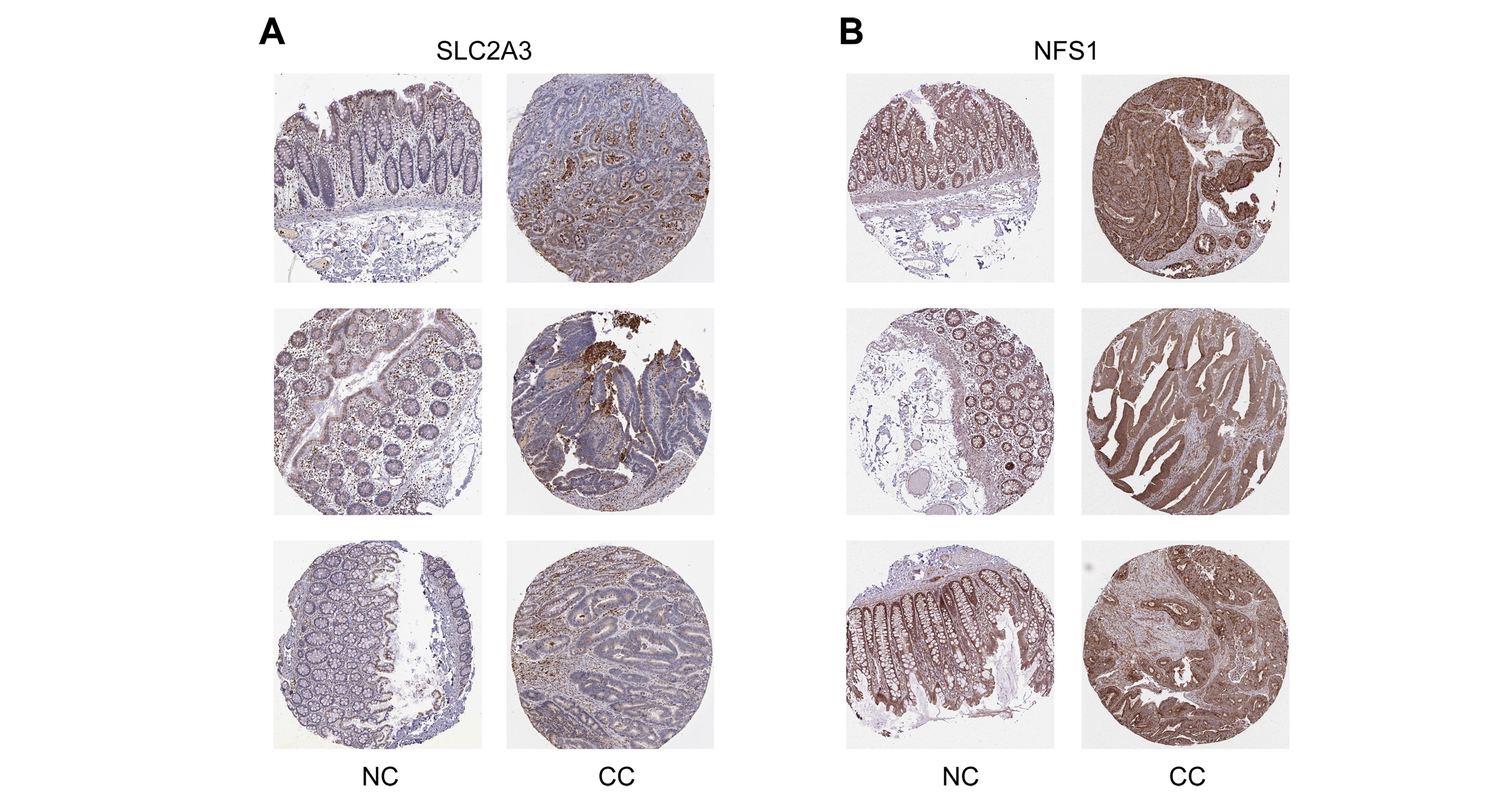

Supplement: Supplementary Figure 3 — Protein expression level of SLC2A3 (A) and NFS1 (B) in normal colon and CC tissues based on immunohistochemistry images from the human protein atlas database. NC, normal colon; CC, colon cancer. [file Image_3.TIF]

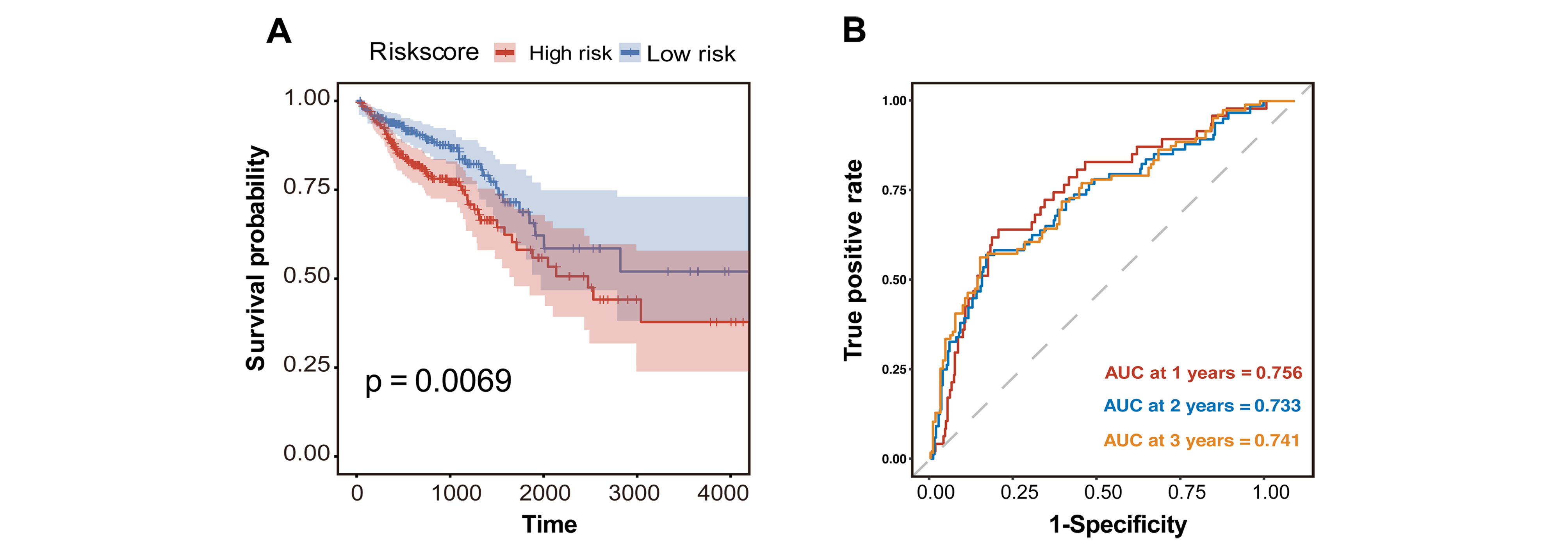

Supplement: Supplementary Figure 4 — Validation of the FRG prognostic signature in the TCGA-COAD dataset. (A) Kaplan-Meier plot for overall survival of CC patients in high- and low-risk groups. (B) Time-dependent ROC curves for the prognostic performance of the FRG signature. It should be noted that the VLDLR gene was filtered out from the signature during Kaplan-Meier survival curve analysis and time-dependent ROC curve analysis due to its low expression level in the TCGA-COAD dataset. [file Image_4.TIF]
